# 

**Published:** 1855-11

**Authors:** 


### CONTENTS

ORIGINAL COMMUNICATIONS—

| | |
|---|---|
| Case of Eczema, | 489 |
| Report on Sanitary characteristics of Chicago, | 495 |

SELECTIONS—

| | |
|---|---|
| On Sterility, treatment and cure, | 504 |
| Foreign Correspondence, | 510 |
| Effects of Poison in the treatment of certain Affections, | 515 |
| On the treatment of Inflamed Breasts, | 516 |

BOOK NOTICES—

| | |
|---|---|
| Diseases of the Rectum, | 517 |
| A Manual of Clinical Medicine, | 518 |
| A Manual of Pathological Anatomy, | 518 |

EDITORIAL—

| | |
|---|---|
| Notice, | 525 |
| Dr. Geo. Coatsworth, | 525 |
| Yellow Fever at Norfolk, | 525 |
| University of Michigan and the Peninsular Journal, | 528 |

